# Synergistic catalytic mechanism of red mud in the co-gasification of spirit-based distillers’ grains and sewage sludge

**DOI:** 10.1038/s41598-024-60434-7

**Published:** 2024-04-26

**Authors:** Xi Zeng, Junliang Wang, Aijiang Yang, Yang Cao

**Affiliations:** 1 Guizhou Guida Yuanheng Environmental Protection Co. Ltd, Guiyang, 550025 China; 2 Institution of Environmental Engineering Planning and Design, Guizhou University, Guiyang, 550025 China; 3 College of Chemistry and Chemical Engineering, Guizhou University, Guiyang, 550025 China; 4 College of Resources and Environmental Engineering, Guizhou University, Guiyang, 550025 China

**Keywords:** Co-gasification, Fixed bed, Catalytic gasification, Disposable catalyst, Energy and society, Sustainability, Renewable energy

## Abstract

Experiments of co-gasification of spirit-based distillers’ grains (SDG) and sewage sludge (SS) were carried out with red mud (RM) by using a self-designed fixed-bed gasifier. The effects of RM addition, gasification reaction temperature, SS and SDG blending ratio and other factors on the gasification reaction characteristics and synergism were investigated. The results are as follow: RM had catalytic effect on SS and SDG co-gasification, which can enhance the gasification reaction and H_2_ yield; increasing the temperature can enhance the gasification reaction and reduce the syngas H_2_/CO; with the increase of SDG, the H_2_ yield gradually grew; with the rise of SS, the gasification reaction gradually augmented. The catalytic mechanism was mainly due to the redox cycle of Fe_2_O_3_ in RM, which can promote the water transfer reaction. At the same time, the eutectic mixture of K, Na, Ca, Fe and other metal elements at high temperatures was the main reason for the synergistic effect.

## Introduction

The rapid development of China’s economy and population have subsequently generated a large amount of solid waste: spirit-based distillers’grains (SDG) are the main by-product of the brewing industry, and the annual production of SDG is more than 100 million tons in China^1^; sewage sludge (SS) is a solid by-product of sewage treatment plants, and approximately 30 million tons (80% water content) are produced annually in China^2^; red mud (RM) is a bauxite smelting waste, which is a solid waste generated in the process of smelting bauxite ore^3^, and the accumulated stockpile of RM has exceeded 1.6 × 10^9^ tons in China^4^. The current treatment of solid waste is mainly simple piling, returning to the field, and composting^5^. There are a large number of organic components in SS and SDG, which is a waste of resources if not resourcefully used.

At present, the energy sources that human relies on are mainly fossil fuels, which account for 87% of global energy consumption^6^, and the greenhouse effect caused by CO_2_ emissions is becoming increasingly serious. Consequently, research on new energy sources is urgent. Many scholars have devoted to the research of new hydrogen production methods^7,8^, as the combustion product of hydrogen is only water, which can achieve zero emission. Gasification can not only effectively reduce the production of toxic substances in solid waste^9^, but also can be used to generate syngas, which is a promising way to treat solid waste.

The SS is more difficult to supply and has less gasification efficiency when gasified alone because it contains higher ash and moisture, yet lower carbon content. By contrast, biomass has lower ash and moisture, but higher carbon content. The prominent complementarity between SS and biomass in physicochemical property and reactivity is conducive to achieve the comprehensive, clean and efficient usage of these two carbonaceous materials. The use of solid waste gasification for hydrogen production has been extensively studied, Ong et al.^10^ conducted an experimental and numerical study of woody biomass and SS gasification by using a fixed bed, and the experimental results showed that 20 wt% of dry SS in the feedstock was effectively gasified to produce syngas containing more than 30 vol% of syngas with an average low calorific value of 4.5 MJ/Nm^3^. Jiao et al.^11^ used SS and pine sawdust for co-gasification and the results indicated that optimal blending ratio of SS was 40 to 60% with the hydrogen content, gas yield and carbon conversion being 40%, 0.70 m^3^·kg^−1^ and 64.1% respectively. However, the co-gasification couldn’t further enhance the gasification reaction and regulate the ratio of syngas, and this defect could be improved by adding additives.

Iron-based catalysts are more widely available, relatively inexpensive, stable, and a better catalyst for gasification^12,13^. RM is rich in iron compounds, and preliminary research has been done at the international level by using it as the gasification catalyst. It was found that RM has good catalytic performance in the gasification process. Yanik et al.^14^ studied the supercritical water vapor gasification of biomass with RM as the catalyst. The results indicated that RM can effectively improve the reactivity of biomass coke and increase H_2_ yield. Zhang et al.^15^ studied the pyrolysis and gasification of cyanobacterial chemical chains based on RM oxygen carriers, and the results indicated that the RM oxygen carriers significantly promote both cyanobacterial pyrolysis and gasification reactions. Zhao et al.^16^ used SDG and RM to catalyze anthracite gasification, and the results indicated that RM and SDG produce synergistic catalytic effects on improving the gasification and hydrogen production efficiency. However, these studies mostly focus on RM for single biomass or coal gasification. In addition, studies on the catalytic activity of RM for co-gasification of two biomasses are still relatively few, and there is no study using RM to catalyze the co-gasification of SS with SDG.

The effects of RM addition, gasification reaction temperature, and SS and SDG blending ratio on the gasification reaction, gas composition and co-gasification synergy were investigated in a fixed-bed gasifier by using SS and SDG. With RM as the catalyst and water vapor as the gasification agent, the synergistic catalytic mechanism was investigated in the present work. The results are useful for improving the efficiency of SS and SDG co-gasification and realizing the industrialization of solid waste resource.

## Materials and methods

### Material

**Figure 1:**
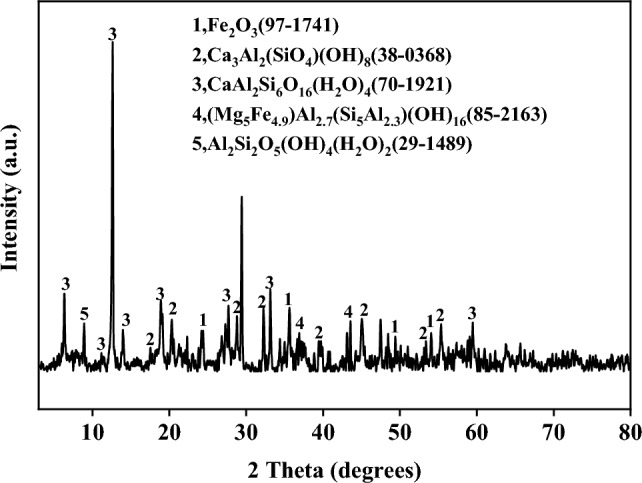
XRD analysis of RM.

SS, SDG and RM coke were from Guizhou wastewater treatment plant, China,Guizhou white wine factory, China and Guizhou alumina plant, China, respectively. The raw materials were crushed and ground, and dried in the particle size range of 58-75 μm. SS, SDG and RM were mixed by using wet method. SS and SDG were added in the amount of 5 g and mixed in a beaker in the mass ratio of 1:0, 3:1, 1:1, 1:3, 0:1, and RM was added in the amount of 12.5%, 25%, 37.5%, 50% of the mass of SS and SDG mixture. Then ultra-pure water was added and stirred magnetically for 24 h, followed by drying in an oven at 68 °C for 48 h. After the samples were completely dried, ground and sealed them for use. The industrial and elemental analyses of the samples used in this paper are shown in Table 1, the X-ray fluorescence spectrometry (XRF) measurements in Table 2, and the XRD analysis of RM in Fig. 1. It can be seen that SS contains higher levels of Ca and SDG has higher levels of K than SS, and that RM is mainly composed of Al, Si, Fe and Ca elements, and the main form of Fe element exists as Fe_2_O_3_.

**Table 1:** Proximate and elemental analysis of samples.

Sample	Proximate analysis, wt%				Ultimate analysis, wt%				
	Mad	Vd	Ad	FCd	C	H	N	S	Other
SS	4.81	31.69	66.84	1.39	12.06	2.27	1.41	0.39	83.87
SDG	9.44	69.47	5.00	16.09	60.1	4.82	3.26	0.23	31.59

**Table 2:** XRF analysis of the raw materials (wt%).

Sample	Al_2_O_3_	SiO_2_	Fe_2_O_3_	TiO_2_	CaO	MgO	Na_2_O	K_2_O	Other
SS	21.70	38.80	7.28	1.35	18.41	4.02	0.224	2.05	6.166
SDG	8.24	52.44	4.08	0.52	7.31	4.85	2.60	8.24	11.72
RM	27.40	14.86	26.07	4.24	10.74	1.59	8.51	4.25	2.34

### Method

**Figure 2:**
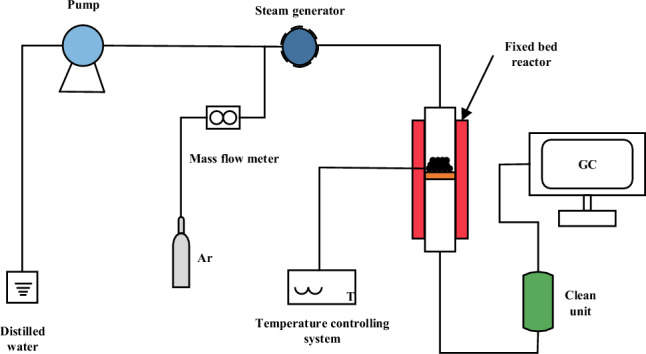
Schematic diagram of the gasification unit.

Steam gasification experiments were carried out in a fixed-bed gasifier. The experimental procedure is shown in Fig. 2. The reactor was a stainless steel tube (length 900 mm, inner diameter 20 mm) and the reactants were placed in the core area of the reactor. The reactor was heated to 700 °C, 750 °C, 800 °C, 850 °C or 900 °C at a rate of 10 °C/min in an argon atmosphere, and then water vapor was introduced. The vapor was supplied by a pump, and after passing through a preheater, it was supplied to the reactor at a flow rate of 0.27 mL/min. The main gas products, H_2_, CO, CH_4_ and CO_2_, were determined by gas chromatography (Aglient 720A), TDX-01 column (3 m × 1/8 inch) and TCD detector (column chamber temperature 185 °C, detector temperature 200 °C) for online quantitative analysis, sampling every 5 min.The reaction was terminated when the concentration of CO reached the lower limit of gas chromatography detection.

### Data processing method

The instantaneous release rate of gasification products, carbon conversion rate and gas release rate are expressed as Eqs. (1–2)

1 $$ r_{i} = \frac{{\frac{{V \times y_{i} }}{{100 - \sum {y_{i} } }} \times 100}}{{22.4 \times m_{0} }}\left( {i = {\text{H}}_{{2}} ,\;{\text{CO}},\;{\text{CH}}_{{4}} ,\;{\text{CO}}_{2} } \right) $$

2 $$ X = \frac{{\int_{0}^{1} {r_{i} \times dt} }}{{\int_{0}^{{{\text{total}}}} {r_{i} \times dt} }}\left( {i = {\text{CO}},\;{\text{CH}}_{{4}} ,\;{\text{CO}}_{2} } \right) $$

where ${\text{y}}_{\text{i}}$ means concentration of H_2_, CO, CH_4_, CO_2_ (v/v%), ${\text{m}}_{0}$ initial carbon mass of the sample (g), ${\text{V}}$ flow rate of the carrier gas argon (mL/min), ${\text{X}}$ carbon conversion rate (%) and ${\text{t}}$ gasification time (min).

The carbon conversion rate (${\text{X}}_{\text{cal}}$) of the mixture is expressed as Eq. (3)

3 $$ X_{cal} = X_{S} \times \left( {1 - P_{B} } \right) + X_{B} \times P_{B} $$

where ${\text{X}}_{\text{S}}$ means carbon conversion of sludge, ${\text{X}}_{\text{B}}$ carbon conversion of lees,$\mathrm{and }{\text{P}}_{\text{B}}$ proportion of SDG in the mixture.

${\text{R}}_{0.9}$ was employed to quantify gasification reactivity.Where T_x=0.9_ means the time when the carbon conversion rate reaches 0.9, R_0.9_ means the reaction index, R_0.9,exp_ and R_0.9,cal_ is the experiment and calculete reaction index respectively.When the synergetic factor is more than 1, it has the catalytic effect, and less than 1, the inhibitory effect.^17^.They are expressed in Eqs. (4)–(5)

4 $$ R_{0.9} = \frac{0.9}{{t_{X = 0.9} }} $$

5 $$ {\text{Synergetic factor}} = \frac{{R_{0.9,\exp } }}{{R_{{{0}{\text{.9,cal}}}} }} $$

## Result and discussion

### Effect of RM on the synergistic gasification of sludge and lees

In this section, the effects of SS and SDG blending ratios on the co-gasification characteristics and synergism were investigated at 800 °C with the variable of 37.5% RM addition, where the SS-to-SDG blending ratios were 1:0, 3:1, 1:1, 1:3, and 0:1, respectively.

**Figure 3:**
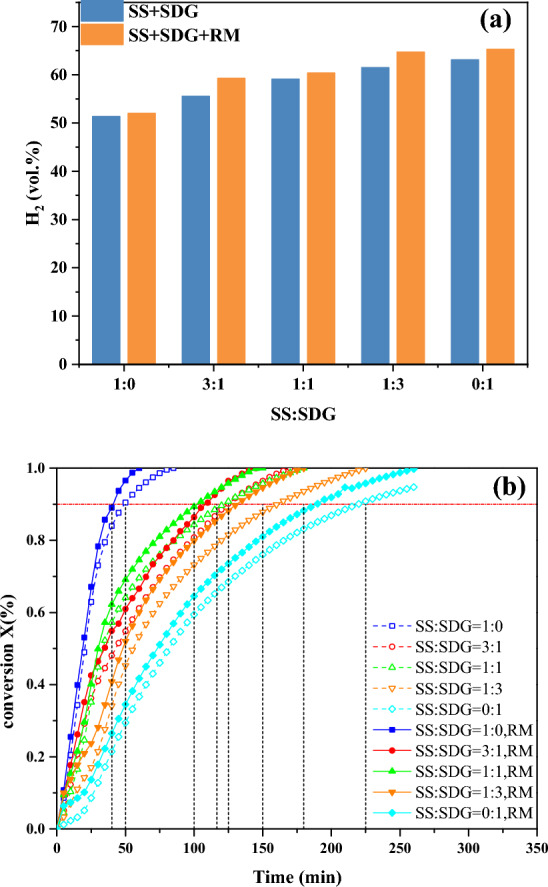
Effect of SS and SDG blending ratio on gas composition and carbon conversion.

The effect of SS and SDG blending ratio on H_2_ content and carbon conversion are shown in Fig. 3a. The H_2_ content gradually increases with the rise of SDG. At any blending ratio, the H_2_ content grows after the addition of RM. In Fig. 3b, the dashed and solid lines show the curves of carbon conversion with time and without and with RM addition, respectively, and it can be seen that the gasification reaction is gradually enhanced and the time is shortend as the SS increases. Hence, SS plays a facilitating role in SDG gasification, mainly because the higher content of metal elements in SS plays a facilitating role in the gasification reaction. After the addition of RM, the time is shortened than that without it, indicating that RM has a catalytic effect on different SS and SDG doping ratios, which promotes the gasification reaction rate and enhances gasification reaction.

**Figure 4:**
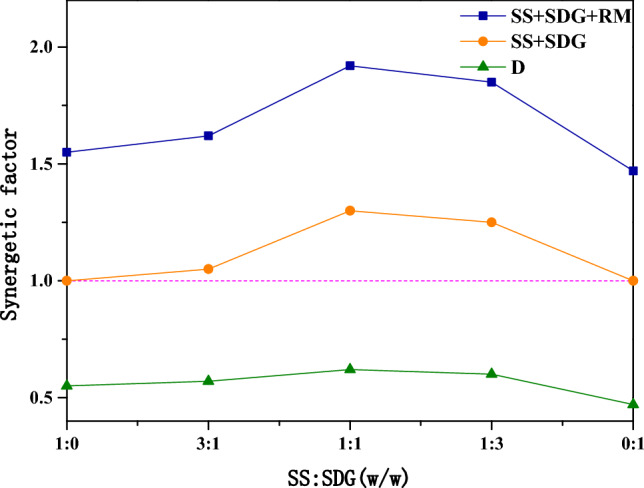
Effect of SS and SDG blending ratio on co-gasification synergy factor.

The synergistic factors for different blending ratios of SS and SDG are shown in Fig. 4. The results show that with the increase of SDG, the cofactor first gradually grows and reaches the maximum at the ratio of 1:1, and then starts to fall when the ratio increases further. Due to the high content of SS ash, when SS is high, its ash will block the inner orifice of SDG and the mass transfer resistance becomes larger, which leads to the lower co-gasification synergistic effect. With the rise of the SDG, the surface area increases and the minerals in SS ash can adhere more to the SDG surface, resulting in a better synergistic effect. However, when the SDG is too high and the SS ash is lower, the catalytic component is lower and the synergistic factor reduces. From the figure, it can be seen that the addition of RM can significantly improve the synergistic factor of SS and SDG co-gasification. Since SS is rich in Ca elements, SDG is rich in K elements, and RM is rich in Fe elements, after the addition of RM to SS and SDG, the synergistic catalysis of metal elements occur, and the synergistic factor is further improved, and the strongest synergistic catalysis is achieved at the ratio of 1:1.

### Effect of co-gasification Temperature

The gasification reaction temperature is an important influencing factor in the gasification process. In this section, the effect of co-gasification was investigated at the SS-to-SDG ratio of 1:1, 37.5% RM addition, and temperatures of 700 °C, 750 °C, 800 °C, 850 °C and 900 °C, respectively.

**Figure 5:**
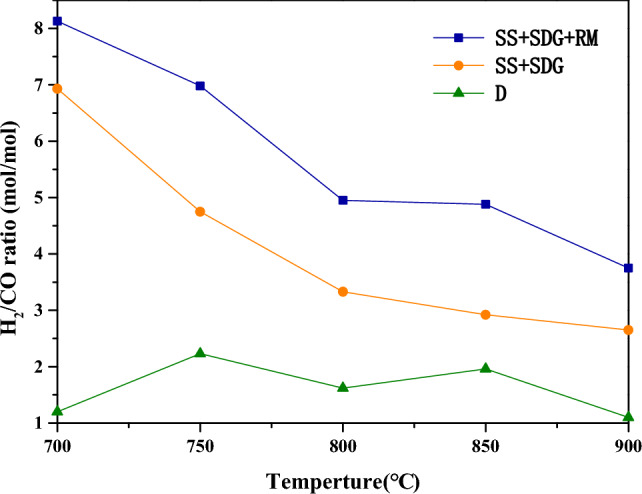
Effect of reaction temperature on H_2_/CO in syngas.

The effect of the reaction temperature on H_2_/CO are shown in Fig. 5. When the reaction temperature is gradually increased from 700 to 900 °C, H_2_/CO ratio shows a decreasing trend. Here are the reasons: the Boudouard reaction and steam gasification reaction are both heat-absorbing reactions, the increase of temperature promotes the production of CO; WGSR(${\text{CO + H}}_{{2}} {\text{O}} \leftrightarrow {\text{CO}}_{{2}} {\text{ + H}}_{{2}}$) is an exothermic reaction; the increase of temperature shifts the reaction in the opposite direction, thus suppressing the production of H_2_ and promoting the production of CO production. With the increase of temperature, the H_2_ concentration decreases and the CO concentration increases, which eventually leads to the reduction of H_2_/CO value. Besides, RM has a catalytic effect on WGSR, which promotes the consumption of CO as well as the production of H_2_. As a result, after the addition of RM, the H_2_/CO ratio is higher than that without RM at all temperatures.

**Figure 6:**
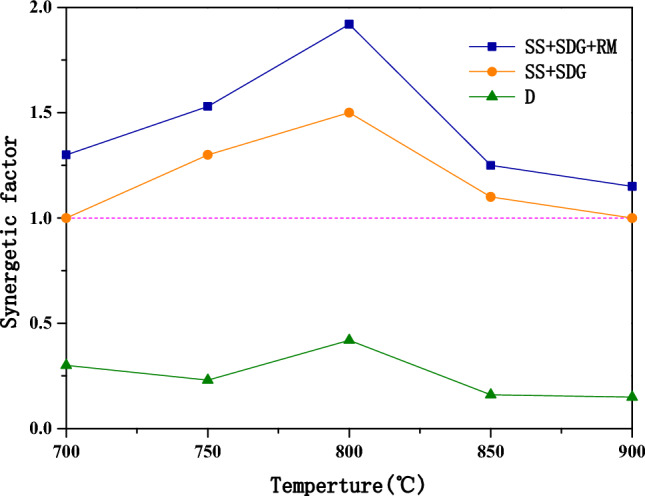
Effect of gasification reaction temperature on the synergistic factor.

The effect of temperature on the gasification cofactor are shown in Fig. 6. The cofactor gradually increases when the temperature increases from 700 to 800 °C, and decreases from 800 to 900 °C. When the temperature is low, the mobility of metal elements is poor, which is not conducive to the migration of metal elements in the materials. Then the synergistic catalytic effect produced by metal elements is poor. When the temperature increases, the migration of metal elements is more active, which can rapidly renew the active sites of gasification reaction, and gradually boost the synergism produced by different metal elements. However, when the temperature is too high, K salts are prone to evaporation, Ca salts are prone to sintering. In additon, K salts are prone to react with clay minerals to produce water-insoluble silicates (e.g., KAlSiO_4_, KAlSi_3_O_8_), which have no catalytic activity^19^. The reactions of Fe_2_O_3_ with H_2_ and CO are also all heat-absorbing reactions, and as the temperature increases, they also promote the the reduction of Fe_2_O_3_. Therefore, when the temperature is too high, the metal element is gradually decreasing due to the deactivation and reaction of the metal element, which eventually leads to the reduction of the co-gasification cofactor. It can be seen from the figure that the co-gasification cofactor reaches a maximum value of 1.92 at the temperature of 800 °C.

### Effect of RM addition on co-gasification

In this section, the effect of RM addition on the performance of SS and SDG co-gasification reaction was investigated. 12.5%, 25%, 37.5%, and 50% of RM were added to the SS and SDG mixture. The temperature was chosen to be 800 °C and the SS-to-SDG ratio was 1:1.

**Figure 7:**
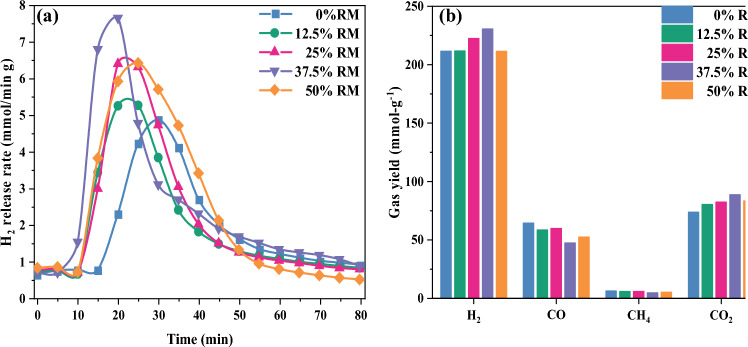
Effect of RM addition on gasification characteristics.

The relationship among RM addition, H_2_ release rate and gas yield are shown in Fig. 7, which can be used to measure the gasification reaction. As shown in Fig. 7a, the maximum value of H_2_ release rate (Rmax) gradually increases as the RM addition gradually grows from 0 to 37.5%, indicating that the gasification reaction gradually augments. When the RM addition continues to increase from 37.5 to 50%, Rmax decreases, indicating that the gasification reaction declines. In addition, RM, as a catalyst, can reduce the activation energy of the reaction, which can make the gasification reaction easier. With the increase of RM addition, the active component gradually increases and the catalytic effect gradually enhances. Consequently, the co-gasification reaction also gradually rises. With the further increase of RM addition, the catalytic effect of RM has reached saturation, and further addition does not play a catalytic role in the gasification reaction anymore. Excessive amount of RM may be deposited on the reactant surface, forming inactive agglomerates. It will occupy the active sites on the reactant surface, resulting in the inability of the gasification agent to react with the reactants effectively and leading to a weakening of the reaction activity^18^. The inert component in RM continues to increase, producing a stronger inhibitory effect on the gasification reaction and eventually leading to a drop in gasification reaction. 37.5% RM addition has the greatest catalytic effect on SS and SDG co-gasification.

It can be seen from Fig. 7b that H_2_ and CO_2_ yields gradually increase and CO yield gradually decreases as the RM addition gradually grows from 0 to 37.5%, indicating that RM promotes the water transfer reaction WGSR, and similar conclusions are obtained by Duman et al^19^ for the catalytic gasification of algae with RM and by Uddin et al^20^ for the catalytic gasification of biomass vapors using Fe_2_O_3_. When RM addition is increased from 37.5 to 50%, H_2_ and CO_2_ production decrease and CO production increases. When the amount of RM is up, the RM oxygen carrier provides more oxygen for the co-gasification, which makes it easier to oxidize the coke obtained from the cracking of SS and SDG to produce CO_2_. When the concentration of CO_2_ increases, it dilutes the concentration of the other gases, promoting the forward progress of various reactions. In addition, RM has a certain positive effect on the cracking of tar, which can produce more small molecule gases. When the amount of RM is further increased, the catalytic substances in RM have reached saturation, and the inert components in RM have strengthened the inhibition effect on the gasification reaction, which ultimately leads to the weakening of the positive effect of RM on WGSR. As can be seen from Fig. 7, when SS-to-SDG ratio is 1:1 and the gasification reaction temperature is 800 °C, the addition of 37.5% RM has the greatest positive effect on the gasification reaction and gas yield.

**Figure 8:**
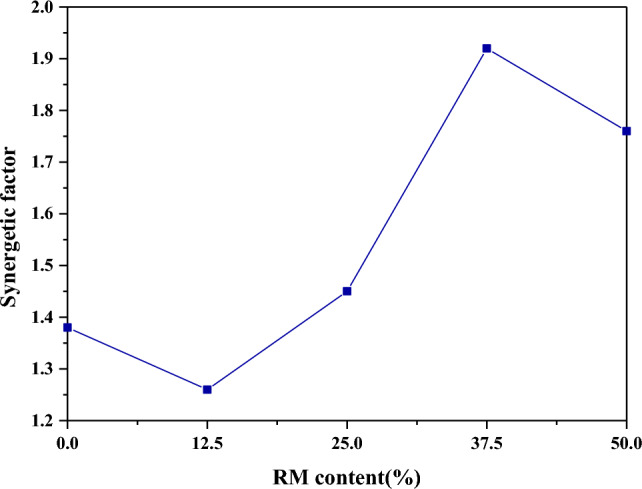
Effect of RM addition on synergistic factors.

The effect of RM addition on the co-gasification cofactor are shown in Fig. 8. The co-gasification cofactor decreases when the additive amount of RM is 12.5% compared with no RM addition; when the additive amount is gradually increased to 37.5%, the cofactor gradually grows, and the co-gasification has the maximum cofactor of 1.92 when the amount is 37.5%; when the amount increases to 50%, the cofactor reduces. Component content is low, and part of Fe_2_O_3_ may react with some components in the reactant ash to produce compounds that are not catalytic, which cannot increase the active sites on the reactant surface in time and have weaker catalytic performance for co-gasification^21^, and the inert components in RM have a stronger inhibition effect on co-gasification, which eventually leads to weaker co-gasification synergistic effect. With the further increase of RM, the content of Fe_2_O_3_, the catalytic property and the synergistic effect gradually enhance. When the additive amount is more than 37.5%, the excessive Fe_2_O_3_ no longer has a catalytic effect on the co-gasification. With the rise of RM, the inert components and the inhibition effect on co-gasification have further increased. Therefore, 37.5% RM addition has the maximum synergistic catalytic effect on SS and SDG.

### Synergistic catalysis and its mechanism

Fe_2_O_3_ is the main catalytically active component in RM, which promotes the WGSR, and the redox cycle process can explain the Fe_2_O_3_ catalytic process. Firstly, Fe_2_O_3_ is reduced by CO, C or H_2_ to form Fe_3_O_4_, and the reduced Fe_3_O_4_ is oxidized by H_2_O to form Fe_2_O_3_. Then, Fe_2_O_3_ promotes WGSR during the redox cycle.

6 $$ 3{\text{Fe}}_{2} {\text{O}}_{3} + {\text{CO}} = 2{\text{Fe}}_{3} {\text{O}}_{4} + {\text{CO}}_{2} $$

7 $$ {\text{3Fe}}_{2} {\text{O}}_{3} + {\text{C}} = 2{\text{Fe}}_{3} {\text{O}}_{4} + {\text{CO}} $$

8 $$ {\text{Fe}}_{2} {\text{O}}_{3} + {\text{CO}} = 2{\text{FeO}} + {\text{CO}}_{2} $$

9 $$ 3{\text{Fe}}_{2} {\text{O}}_{3} + {\text{H}}_{2} = 2{\text{Fe}}_{3} {\text{O}}_{4} + {\text{H}}_{2} {\text{O}} $$

10 $$ {\text{3FeO}} + {\text{H}}_{2} {\text{O}} = {\text{Fe}}_{2} {\text{O}}_{3} + {\text{H}}_{2} $$

11 $$ 2{\text{Fe}}_{3} {\text{O}}_{4} + {\text{H}}_{2} {\text{O}} = 3{\text{Fe}}_{2} {\text{O}}_{3} + {\text{H}}_{2} $$

**Figure 9:**
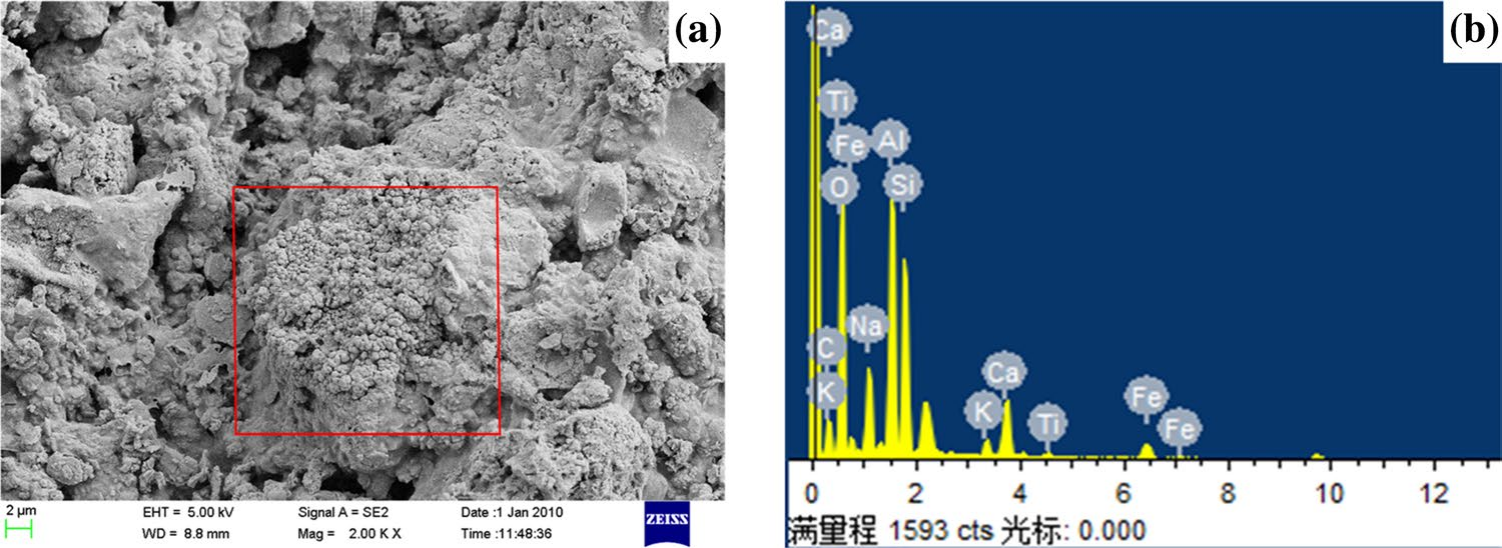
SEM–EDS analysis of SS/SDG/RM gasification reaction rate at maximum.

In order to investigate the synergistic catalytic effect produced by RM co-gasification with SS and SDG, SEM–EDS analysis was performed on the residual carbon at the time when Rmax was reached in the co-gasification at the SS-to-SDG ratio of 1:1, with 37.5% RM addition and at the temperature of 800 °C, as shown in Fig. 9. From Fig. 9a, it can be seen that a large amount of eutectic mixture is formed on the surface of the residual carbon at high temperatures. Figure 9b shows the EDS analysis of this eutectic mixture. The results show that the mixture contains metal elements such as Fe, K, Na, and Ca. SS and SDG are rich in alkali metals K and Na, which have better migration characteristics under thermal conditions, thus distributing well on the carbon matrix surface and activating the carbon matrix, which in turn generates active centers that can react directly with water vapor to generate H_2_^22,23^. Fe and Ca, due to their higher melting temperature and poor mobility, affect the gasification reaction rate mainly by changing the pore structure of the biomass. When K, Na, Ca, and Fe form a composite catalyst, the melting point is lower than that of a single component catalyst. Besides, the produced eutectic mixture has good mobility. It can increase the contact area with the biomass surface,and form a catalytic active central site. As a result, it can better promote reaction than a single catalyst and the addition of RM can have a synergistic catalytic effect on SS and SDG co-gasification^16^. Gao et al^24^ also found that Na has good mobility and will form a bimetallic mixture Na_2_Ca(CO_3_)_2_ with Ca during the gasification process, which can inhibit the agglomeration of CaO and thus produce a synergistic effect.

## Conclusions

The effects of RM addition, reaction temperature, SS and SDG blending ratio on the reaction activity, produced gas composition and synergistic effect of SS and SDG co-gasification were studied by using a fixed-bed reactor, and the following conclusions were obtained:

1. RM had a good catalytic effect on SS and SDG co-gasification, and its additive amount had a large effect on the gasification reaction. 37.5% RM addition had a significant improvement on the gasification reaction and H_2_ yield, and the synergistic effect was optimal.
2. The effect of temperature on the gasification reaction was obvious. As the temperature increased, the H_2_/CO and the gasification reaction gradually enhanced. The synergistic factor gradually grew when the temperature was increased from 700 to 800 °C, and reduced at temperature rising from 800 to 900 °C. When the temperature was low, the metal ions in RM were not active enough. The cofactor increased with the rise of temperature and reached the optimum at 800 °C.
3. The H_2_ yield increased gradually with the rise of SDG, and the gasification reaction augmented with the increase of SS. With 37.5% RM addition, at 800 °C gasification reaction temperature and SS-to-SDG ratio of 1:1, the synergistic factor reached the maximum value of 1.92.
4. The active component in RM promoted the WGSR reaction, thus improving the H_2_ yield, and the catalytic mechanism was redox cycle. SEM–EDS analysis was performed on the residual carbon, it can be seen that a large amount of eutectic mixture is formed on the surface of the residual carbon. The synergistic effect was mainly generated by the eutectic mixture of K, Ca, Na, and Fe, which had good mobility, and then better had catalytic effect on gasification.

## Data Availability

All data generated or analysed during this study are included in this published article.
